# Conversational topic maintenance and related cognitive abilities in autistic versus neurotypical children

**DOI:** 10.1177/13623613241286610

**Published:** 2024-10-21

**Authors:** Kirsten Abbot-Smith, Danielle Matthews, Colin Bannard, Joshua Nice, Louise Malkin, David Williams, William Hobson

**Affiliations:** 1 University of Kent, UK; 2 University of Sheffield, UK; 3 University of Manchester, UK

**Keywords:** ADOS, conversation, non-contingent, off-topic, school-age children, theory of mind, verbosity, working memory

## Abstract

Keeping a conversation going is the social glue of friendships. The *DSM* criteria for autism list difficulties with back-and-forth conversation but does not necessitate that all autistic children will be equally impacted. We carried out three studies (two pre-registered) with verbally fluent school children (age 5–9 years) to investigate how autistic and neurotypical children maintain a conversation topic. We also investigated within-group relationships between conversational ability and cognitive and sociocognitive predictors. Study 1 found autistic children were more likely than neurotypical controls to give off-topic and generic minimal responses (e.g. ‘mm’, ‘oh’) and were less likely to give non-verbal responses (e.g. nodding or use of facial affect to respond). Nonetheless, the autistic group provided topic-supporting responses 62% of the time, indicating some aspects of conversation topic maintenance are a relative strength. Studies 2 and 3 found large individual differences in topic-supporting conversational responding among both neurotypical and autistic children. These were positively related to theory of mind ability and age in both groups. Conversational skills lie on a continuum for the general population and differences by diagnostic group are a matter of degree. Given the importance for peer relationships, we suggest a whole classroom approach to supporting conversation skills in all children.

**Lay abstract:**

Children who struggle to maintain conversation with peers often have fewer friends and lower popularity ratings, which can affect wellbeing. Verbal social communication more broadly is linked to both behavioural difficulties and emotional problems. We carried out three studies to examine children’s ability to provide responses which keep a back and forth conversation going. The first study found that while autistic children had on average greater difficulties than their neurotypical peers with certain aspects of conversation topic maintenance, for other aspects the autistic group showed considerable strengths. Both studies 2 (neurotypical children) and 3 (autistic children) found relationships between, on the one hand, conversational ability, and on the other, the ability to consider another’s viewpoint and the ability to maintain and update information in short term memory. We suggest support for social conversation skills should be part of mainstream classroom curricula for autistic and neurotypical children alike.

## Introduction

One of the two symptom domains of the diagnostic criteria for autism entails ‘persistent deficits in social communication and social interaction’ manifested by difficulties with social-emotional reciprocity, non-verbal communication and with developing and maintaining relationships (American Psychiatric Association, 2013). Particular hurdles within the first category – social-emotional reciprocity – are specified in the *DSM*-5 to include difficulties with social approach, with back-and-forth conversation, reduced sharing of interests, emotions or affect and failure to initiate or respond to social interactions (American Psychiatric Association, 2013). Thus, the ability to maintain a conversation topic is not only highlighted in the diagnostic criteria but is also critical for most of the other points listed in the domain of social interaction and social communication since conversation skills are central for establishing and maintaining relationships (Friedman et al., 2019). The ‘gold standard’ assessment tools for autism (ADOS-2, ADI-R) involve direct and indirect assessment of conversational ability. Indeed, many have noted that the most common communication deficits seen in intellectually able autistic youth pertain to conversation (Paul et al., 2009).

Yet, to date, there has been a lack of clarity around a) the distribution of conversational skills in neurotypical school-aged children (i.e. those with no diagnoses or suspected disorders) and how this overlaps with that of autistic children and b) the cognitive and social-cognitive skills that are correlated with conversation skill for both groups of children. This is because there are few quantitative studies that have used direct measurements to examine individual differences in how neurotypical children fare with maintaining conversation (see Abbot-Smith et al., 2023 for a review).

To keep a conversation topic going, speakers need to be able to do two key things. First, a speaker needs to be able to provide ‘topic-supporting’ responses. These include ‘contingent responses’, that is, those which provide information that is both relevant to the immediately preceding conversational turn and add information (Bloom et al., 1976; Pagmar et al., 2022). Another way to support a conversation topic is to provide ‘specific’ minimal responses (e.g. ‘*Really?*’) or even in certain contexts non-verbal responses, such as laughing, gestures or affective reactions (e.g. an expression of shock) (Bavelas et al., 2000). All of these responses indicate that one has processed and is interested in what the speaker is saying. In contrast, non-contingent (off-topic) responses can provide a stumbling block for conversation topics (Hale & Tager-Flusberg, 2005; Nadig et al., 2010). Children in upper primary school and adolescents – both autistic and neurotypical – give significantly lower ratings of intentions to interact socially with children they have heard responding non-contingently (McGuinness et al., in press; Place & Becker, 1991). An example of a non-contingent response from our dataset is as follows:

RESEARCHER: ‘I like cooking biscuits with chocolate chips in them. They’re my favourite’.

P2: ‘There’s twins in my class. They’re identical’.

The second key conversational ability required to maintain a conversation topic involves restricting the length of each turn to avoid ‘monologuing’ (i.e. talking excessively without allowing the partner a turn). While non-contingency and excessive verbosity have often been discussed in relation to autism (Cola et al., 2022; Nadig et al., 2010; Ying Sng et al., 2018), it is unclear to what degree autistic children as a group show conversational impairments, particularly now that far more verbally fluent autistic children are receiving autism diagnoses (e.g. McConkey, 2020).

Moreover, there are large individual differences among neurotypical children in the ability to maintain a conversation topic (see, e.g. De Rosnay et al., 2014 for 5- to 8-year-olds). Conversational contingency relates to sociometric ratings by peers in 4-year-olds (Hazen & Black, 1989) and training conversation skills leads to higher rates of playground social interaction in neurotypical 10- and 11-year-olds (Bierman & Furman, 1984). Even among neurotypical adults, conversational ability relates to ratings (by others) of attraction (Wheeless et al., 1992) and interpersonal relationship satisfaction (Miczo et al., 2001). Indeed, as it has been demonstrated experimentally that adults rate other adults less positively on scales of likeability when the latter are less conversationally responsive (Mein et al., 2016). Given the implications for lifelong social relations, we need a better understanding of the degree of heterogeneity in conversational ability within both populations (autistic and neurotypical) and we also need to know the degree to which conversation skills show distinct relationships with key cognitive abilities in each group.

The ‘key suspects’ in the literature regarding the cognitive underpinnings of conversational ability are theory of mind and/or executive functioning abilities such as working memory or inhibitory control (see Matthews et al., 2018 for a review). There are some studies which have examined relationship between either theory of mind or executive functioning and parent questionnaire measures of pragmatics or conversation (Baixauli-Fortea et al., 2019; Hutchison et al., 2020). However, there are very few studies that have examined either theory of mind or executive functioning in relation to *directly* elicited child conversation. Nonetheless, of those that have, there is evidence of links between contingent responding and theory of mind in both autistic primary-school-aged children (Hale & Tager-Flusberg, 2005) and neurotypical children (Slomkowski & Dunn, 1996). However, the degree to which theory of mind uniquely accounts for contingent responding is not clear given that individual differences in theory of mind itself are correlated with both working memory and inhibitory control (Fizke et al., 2014; Lecce et al., 2017). Furthermore, the one prior study that examined relationships between direct measures of child conversation and executive functioning found that working memory (Backwards Digit Span) correlated positively with contingent responding and ‘fluidity’, whereas inhibitory control correlated negatively with both child talkativeness and number of words per utterance (Blain-Brière et al., 2014). There is therefore a question as to whether any given candidate cognitive underpinning explains unique variance when other candidates are controlled for.

Blain-Brière et al.’s (2014) finding of a relationship between inhibitory control and talkativeness speaks to the second key ability required to maintain a reciprocal conversation, namely that it should involve both conversation partners (as opposed to monologuing). However, talkativeness per se is not an inherently negative trait (Coplan et al., 2011). Talkativeness is considered ‘excessive’ or ‘verbose’ – and leads to negative conversation satisfaction ratings – when speakers also tend to veer off-topic during the turn (Pushkar et al., 2000; Ruscher & Hurley, 2000). The only prior study which has directly examined ‘problematic verbosity’ in children is that of Nadig et al. (2010), who termed this ‘self-contingent elaboration’ and found certain group differences between autistic and matched neurotypical 10- and 11-year-olds. However, while a small number of verbally fluent, cognitively able autistic children are ‘excessively verbose’, this is not necessarily a general characteristic of this group (Adams et al., 2002).

In this article, we report on studies with children of primary school age, all of whom had non-verbal reasoning and vocabularies within the neurotypical normal range for their age. In Study 1, we investigate differences and similarities between autistic and neurotypical (NT) primary school children in their ability to maintain a conversation. In Studies 2 and 3, we investigate individual differences within a group of neurotypical primary-school children and within a group of autistic primary school children. Here we also ask whether theory of mind and working memory (and – for one study – inhibitory control) account of individual differences conversation topic maintenance where all predictors are included in the same regression model.

We carried out three studies, all with children tested in southern England. (There was no overlap in participants between any of the studies). Each child was assessed individually in verbal interaction with a psychology researcher or clinician. In the first study, we compared autistic 5- to 7-year-olds with neurotypical children matched for age, non-verbal reasoning, core language and sex. In the second study, we elicited extended naturalistic conversations from neurotypical 6-year-olds and also assessed their theory of mind, working memory, inhibitory control, vocabulary and non-verbal reasoning abilities. In the third study, conversation was elicited by a clinical psychologist (a co-author on this article) from 5- to 9-year-olds who went on to receive an autism diagnosis from a multi-disciplinary assessment in the British National Health Service (NHS). The same children were also assessed on theory of mind, working memory, vocabulary and non-verbal reasoning tasks. The second and third studies were pre-registered prior to the completion of transcription and prior to start of data analysis: https://osf.io/q7wa4/registrations.

## Ethical and sampling statement

Ethical approval was obtained from the first author’s institution for all three studies. In addition, for the third study, NHS ethical approval was also obtained. All parents provided full written informed consent. Socioeconomic circumstances^1^ and ethnicity were not recorded. The adult autism community was not involved in this study.

## Study 1

### Method

#### Participants

We included 30 autistic (7 female) and 30 neurotypical (7 female), monolingual British-English-speaking 5- to 7-year-olds (see Table 1 for demographics). The sample size was agreed between the lead, second and fifth authors prior to data collection as the maximum sample size manageable since the data were collection alongside the fifth author’s final PhD project between January 2019 and March 2020, when the UK COVID-19 lockdown commenced. No participant had hearing difficulties or ADHD. All autistic children had a diagnosis requiring multi-disciplinary consensus within the British National Health Service. All scored above the threshold for autism on the parent-completed Social Responsiveness Scale 2 (SRS) (Costantino & Gruber, 2005). Of the autistic sample, 37% were recruited via an autism charity. The rest were recruited via their school. Of the NT sample, 87% were recruited via schools and only one scored above threshold (T-score of 62 – mild range) on the SRS-2.

**Table 1.** Means (*SD* in parentheses) for participant characteristics.

	Autistic (*n* = 30)	NT (*n* = 30)	*p*	*d*
	Mean (*SD*)	Mean (*SD*)		
Gender split	23 M; 7 F	23 M; 7 F	-	-
Chronological Age (Months)	77.37 (10.58)	76.94 (9.08)	.87	0.04
Sentence Comprehension CELF-5 Scaled Score	9.97 (2.46)	10.2 (1.94)	.68	0.10
Expressive Vocabulary CELF-4 Scaled Score	8.87 (2.70)	9.13 (1.78)	.65	0.11
Non-Verbal Reasoning: BAS Matrices T-Score	41.53 (7.82)	38.77 (7.06)	.16	0.37
Social Responsiveness Scale T-score	84.57 (7.72)	44.37 (6.16)	< .001	5.76

Groups were matched on chronological age, sex, non-verbal reasoning (assessed by the ‘Matrices’ sub-test of the British Ability Scales; Eliot & Smith, 2011), vocabulary as assessed by both the ‘Expressive Vocabulary’ sub-test of the *Clinical Evaluation of Language Fundamentals 4* (Semel et al., 2006) and sentence comprehension ability as assessed by the ‘Sentence Structures’ sub-test of the CELF-5 (Wiig et al., 2013).

#### Procedure

In this study, continuous conversation was not elicited. Instead, the experimenter (the fifth author) uttered pre-planned statements (probes) such as ‘*I like to eat fruit*’ interspersed between standardised tests or games. There were 16 such statements, organised by topic into groups of four (Appendix A). The first statement of a block related to a task or game the child had just completed. The immediate child responses to these 16 conversation probes were video- and audio-recorded and analysed.

#### Data exclusion

If the child did not respond verbally, but the experimenter did not leave at least 2000 ms between the offset of her pre-planned statement and the onset of her following conversational statement, then this item was excluded from analyses. Furthermore, if the experimenter deviated from the syntax of the planned conversational probe, this item was excluded from analyses.

#### Coding conversational responses

For all conversation probes which met the inclusion criteria (see above), each child’s response was coded as either ‘verbal response’ or ‘no language used’. An explanation of the coding scheme, together with some example participant responses to the probe ‘I have a pet’, is outlined in Table 2.

**Table 2.** Conversation coding scheme.

	Coding category	Definition	Example response from Study 1 data
Sentential response	Contingent Responses	Multi-word utterances which are relevant and which elaborate on the content of the conversation partner’s turn (Bloom et al., 1976)	P12: ‘I’ve got 2 pets’ P18: ‘Is it a tiger?’
	Non-contingent Responses	Either (i) were not on the topic of (or only very tangentially related to) the immediately preceding turn, ii) had a very unclear meaning or iii) were ‘bizarre’ or iv) switching to talk about something in the environment (Nadig et al., 2010)	P51: ‘Yeah there’s a new boy and he punched him like some chocolate and he went [NOISE] in the face
Minimal response	Specific minimal responses	Were either (i) one word utterances which were clearly specific to the content of the preceding turn (e.g. E: ‘I have a pet’ C: ‘(A) Doggy?’) or ii) short phrases which encouraged the conversation partner to keep talking (e.g. ‘Did you?’, ‘Was it?’) but which did not elaborate on the topic (Bavelas et al., 2000)	P15: A doggy! P31: What? P28: Have you? (Other possible responses: ‘Really?’, ‘Cool!’)
	Generic minimal responses	Were single words or short phrases which might be acknowledgements of the conversation partner’s turn but which could potentially be uttered without the speaker listening to or processing the conversation partner’s turn (Bavelas et al., 2000; Jefferson, 1984)	P13: ‘Mm-hmm’ (Other possible responses: ‘Yeah’ ‘Oh’ ‘Uh-huh’ ‘Right’)
No language used	Non-verbal Responses	Non-verbal behaviour which acknowledge or respond to the conversation partner’s turn	Nods Raises eyebrows Smiles in response
	Null response	The child either looked at something in the room or else looked at the experimenter’s face with no discernible reaction	

We also included repetitions (partial or full) in with ‘generic minimal’ – although these can serve a communicative function (Luyster et al., 2022) – since there were only four repetitions in our whole dataset.

#### Inter-rater reliability (IRR)

The verbal responses for 23.3% of the data were second coded by a native English-speaking Psychology student, who was ‘blind’ to both the ratings of the main coder as well as to the diagnostic status of the child. There was excellent agreement between the coders (*k* = .92). For non-verbal behaviours, 30% of the dataset was second coded in the same way and reliability was good (Cohen’s *k* = .83).

### Results

The full anonymised dataset is available on the Open Science Framework (OSF) here https://osf.io/q7wa4/. The mean proportion of each type of conversational response is shown in Table 3.

**Table 3.** Mean (*SD* in parentheses) proportion of responses to conversational probes (see Table 2 for definitions).

		Autistic Mean (*SD*)	NT Mean (*SD*)
Sentential response	Contingent	.484 (.5)	.565 (.496)
	Non-contingent	.135 (.342)	.042 (.202)
Minimal response	Specific	.120 (.33)	.116 (.32)
	Generic	.097 (.30)	.038 (.19)
No language used	Non-verbal responses	.015 (.121)	.078 (.268)
	Null response	.145 (.352)	.15 (.356)

We fitted a mixed-effects multinomial logistic regression model using STAN via *brms* (Bürkner, 2017) with the default priors. The six response types (contingent, non-contingent, specific minimal, generic minimal, non-verbal response, null response) were the outcome variables, with null response being the reference response. Diagnostic group (Autistic vs Neurotypical) was included as an effect-coded fixed effect and participant and item were included as random effects on the intercept.

**Figure 1.**
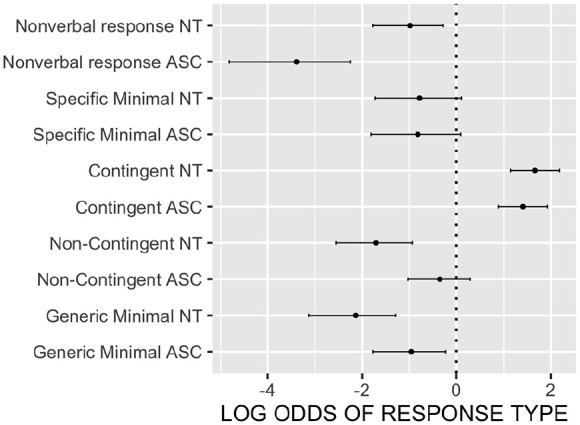
Log odds of each response (relative to the odds of producing no response) for the two participant groups with 95% credible intervals.

Figure 1 shows the estimated log odds ratios for each of the responses for each group, relative to the odds of producing no reaction. The dot for each response/group represents the best estimate for the log odds of that response in that group. The bars represent the uncertainty around this estimate – the range in which the value falls with 95% probability. The vertical dashed line represents the log odds of producing no response. Only the estimates for contingent responding are greater than the estimates for not responding. Critically for each response type, where the best estimate for one group falls outside the interval for the other, we can say that there is < 0.05 probability (two-tailed) that that response has the same likelihood of occurrence for that group as for the other. This is the case for three of the six response categories, with neurotypical children showing lower rates of non-contingent (off-topic) responses (p_mcmc < 0.005) and generic minimal responses (i.e. where it is difficult to know for sure that the child processed the content) (p < 0.05) than autistic children, but higher rates of non-verbal responses such as nodding and/or smiling in response (p_mcmc < 0.0005).

Thus, overall the findings are mixed. The three aforementioned significant differences between groups fit with the diagnostic criteria and with previous literature (e.g. Nadig et al., 2010). However, Table 3 and Figure 1 reveal striking similarities between groups in their ability to use contingent responses and specific minimal responses to keep a conversation topic going.

To further explore this skill among the autistic group, we then followed the method in Pagmar et al. (2022) in conflating over the three response categories which could be termed ‘*topic-supporting’* (which Pagmar et al., 2022, refer to as ‘appropriate’) in a conversation, namely contingent responses, specific minimal responses (see Table 2 for definitions) and non-verbal responses. For this conflated ‘overall topic-supporting responses’ category, we built a binary logistic regression model and found that there was a significant between-groups difference (Est = 0.85, *SE* = 0.34, *z* = 2.516, *p* = .012), largely driven by lower rates in the autistic group of non-verbal responding (i.e. gestures or use of facial affect to respond). However, it is important to highlight that our sample of young primary-school-aged autistic children gave topic-supporting responses to 62% of the conversation probes, indicating that even at this young age, verbally fluent autistic children are able to respond in a way that maintains a conversation topic a large proportion of the time.

### Interim discussion

Although for many of conversational response types, the above findings are consistent with claims (and diagnostic criteria) that highlight conversational weaknesses in autistic children, for other response types there is notable overlap between the diagnostic groups. Moreover, we noticed high variability in both populations. In Study 2, we explored these individual differences in neurotypical children further by investigating the cognitive correlates of conversation ability. We also elicited full conversations as opposed to responses to conversation probes. We then explored individual difference in autistic children in Study 3.

## Study 2

### Method

#### Participants

The study included 48 neurotypical, monolingual British-English-speaking children (24 female), aged between 5;11 and 6;11 years (M = 77.56 months/6;5 years, *SD* = 3.77), recruited via and tested in the Kent Child Development Unit between July 2017 and February 2018. All parents reported that their children had no known or suspected hearing, learning or language difficulties and none had been referred for any neuro-developmental disorders. The sample size was originally planned to be 75 but had to be reduced to 48 due to competing constraints on participant availability (i.e. external funding was obtained by another researcher in the department).

#### Elicitation of conversation

Conversation was elicited by one of two research assistants (RAs), first in relation to one or more of the child’s interests and then on one or more of the specific topics of siblings, pets and families. The RAs (female Psychology graduates in their early twenties) were trained to avoid using a questioning style. The RAs prepared two or three declarative statements per conversation to use at topic-relevant moments. The majority of conversation was unplanned and free-flowing (see Appendix 2 for details).

#### Additional measures and procedure

First, *Expressive Vocabulary* was assessed using a sub-test of the CELF-4 (Semel et al., 2006). Then, *Theory of Mind (ToM)* was assessed via the following commonly administered vignettes: ‘Birthday Surprise’ (Sullivan et al., 1994), ‘Robots’ (Coull et al., 2006), two of Happé’s (1994) strange stories – lying to persuade (‘Kittens’) and Malkin et al.’s (2018) version of ‘double-bluff’. For the ToM verbal responses, 14.6% of the data were coded by a second coder, who was blind to scores of the main coder (Cohen’s *k* = .96). For analyses, we summed across all ToM measures.

After this first bank of tests, the first conversation was elicited following the procedure above. This was followed by a second bank of tests. *Inhibitory Control (IC)* was assessed via a child-friendly version of the Flanker task (Blakey et al., 2020), where the measure is the difference between mean reaction times in the incongruent and congruent conditions. *Working Memory* was assessed using the Backwards Digit Span sub-scale of the Wechsler Intelligence Scale for Children (WISC) (Wechsler, 2003). Then, a second conversation was elicited. Finally, *Non-verbal Reasoning* was assessed using the ‘Matrices’ sub-test BAS-3 (Eliot & Smith, 2011). Of the predictors, only vocabulary was significantly correlated with other predictors, namely age (*r* (45) = .34, *p* = .021), and Theory of Mind (*r* (45) = .37 *p* = .013).

All except one parent completed four sub-scales of the Children’s Communication Checklist 2 (Bishop, 2003), namely D (Coherence), E (Inappropriate Initiation), *F* (Stereotyped Language) and G (Context) in the waiting room during the testing session.^2^

#### Conversation coding and inter-rater reliability (IRR)

Child immediate responses to experimenter turns were coded by the first author using the categories used for Study 1. Since Studies 2 and 3 elicited conversation (as opposed to merely a response to one statement), here we also coded (following Nadig et al., 2010) child utterances that followed on from their own prior utterance as ‘elaborations’ (see details below). Five children (i.e. 390 child utterances) were second coded by a Linguistics Masters student blind to the original coding (Cohen’s *k* = .88).

### Results

We followed a pre-registered analysis plan (https://osf.io/q7wa4/registrations) to first explore children’s responses to experimenter declarative statements and, second, to explore children’s elaborations on their own prior turn.

### Child direct responses to experimenter turns

#### Distribution of conversational response types

Following our pre-registered analysis plan, we focused on the analyses of the following contrasts:

a) overall *topic-supporting* responses (the sum of contingent responses, specific minimal responses and non-verbal responses) as opposed to responses which are either a hurdle for the topic or do not particularly support it (non-contingent, generic minimal responses, null responses);
*b) contingent* responses as opposed to all *other* response types (i.e., non-contingent, all minimal and all non-verbal and null responses);
*c) non-contingent* responses as opposed to all *other* response types (i.e., contingent, all minimal and all non-verbal and null responses).

For comparability with Study 1, mean proportions and mean raw utterances for all fine-grained coded categories of direct responses to an experimenter declarative statement (as well as the summed measure of overall topic-supporting responses) are provided in Table 4.

**Table 4.** Study 2 (neurotypical) child direct responses to experimenter turns, showing the mean proportions, mean raw utterances by child (SD are shown italicised in brackets).

Superordinate	Utterance type	Mean prop (*SD*)	Mean raw *(SD)*
Topic-supporting	Contingent	0.51 *(0.18)*	23.75 *(10.19)*
	Specific minimal	0.09 *(0.07)*	4.54 *(3.86)*
	Non-verbal	0.09 *(0.09)*	4.17 *(3.89)*
Overall topic-supporting responses to experimenter turns		0.69	32.46
	Non-contingent	0.06 *(0.05)*	2.94 *(2.35)*
	Generic minimal	0.14 *(0.11)*	7.38 *(6.76)*
	Null response	0.10 *(0.11)*	4.46 *(5.11)*

#### Investigating the role of cognitive and socio-cognitive predicators

For each of the three outcome variables (overall Topic-supporting responses, Contingent and Non-contingent), we built mixed-effects logistic regression models with Age, Expressive Vocabulary, Theory of Mind, Inhibitory Control, Working Memory and Nonverbal Reasoning as predictor variables (converted to z scores) and participant as a random effect on the intercept. The *p* value for each predictor was calculated by using likelihood ratio tests to compare the full model to a model without that particular predictor. For these analyses below, due to missing data for some predictor variables, the sample size was 39 because non-verbal reasoning scores were missing for five participants, vocabulary scores were missing for an additional three participants and the incorrect Flanker task version was used for an additional participant. (However, zero-order correlations included the full sample, where possible, see Appendix 4).

For overall topic-supporting responses, the only predictors to explain unique variance were age (b = 0.28, χ^2^ = 6.11, *p* = .01) and the theory of mind composite (b = 0.27, χ^2^ = 4.43, *p* = .04), which were both positively related.^3^ For contingent responses, the same pattern was found, where both age (b = 0.48, χ^2^ = 11.11, *p* < 0.001) and theory of mind (b = 0.33, χ^2^ = 4.52, *p* = .03) were positive predictors.^4^

For non-contingent responses, the only predictors to approach significance were the theory of mind composite (b = −0.30, χ^2^ = 3.29 *p* = .07) and working memory (b = −0.26, χ^2^ = 2.92, *p* = .087), such that as these skills increased, non-contingent responses became less likely.^5^

Although non-contingent responses were a relatively infrequent utterance category (see Table 3), the individual differences in their usage deserve highlighting. On the one hand, 17% of these children never gave a non-contingent response. Conversely, 30% of the children gave non-contingent responses to an experimenter turn in more than 10% of their responses. Children’s non-contingent responses tended to either involve thinking of a more interesting topic (see P2 in the section Introduction), becoming distracted by the environment (P13 below) or returning to a previous topic (P4 below):

RESEARCHER (R): ‘He’s like a fish’.

P13: [REFERRING TO HER SHOES] ‘I’m trying to do with these over a bit so they’re a bit tighter’.

RESEARCHER : ‘So all his powers were gone and he couldn’t do anything about it’. [TALKING ABOUT BATMAN]

P4: ‘I might do that with toothpaste and sugar’. [RETURNS TO PREVIOUS TOPIC ABOUT PLAYING PRANKS ON SIBLINGS]

### Child elaborations on their own preceding turn

In addition to responding to researcher turns, children frequently elaborated on their own prior turn. Following Nadig et al. (2010, p. 2735), we categorised elaborations into ‘contingent’ elaborations and ‘self-contingent elaborations’. Contingent elaborations involved extended talk which stayed on the sub-topic established by the experimenter. The other two categories were self-contingent and non-contingent elaborations. Self-contingent elaborations were utterances where the child started (during his/her extended response) to veer off from the topic of the experimenter’s last utterance. Non-contingent elaborations involved the child switching from (her own) topic to another. Some illustrations from the dataset are provided in the coding manual (on OSF https://osf.io/q7wa4/).

#### Distribution of child ‘conversation elaboration’ types

Table 5 indicates the mean proportion and mean raw frequency of each sub-type of child elaboration (follow in) on his/her own response.

**Table 5.** Study 2 (neurotypical) child elaboration type, showing the mean proportion and mean raw utterances by child (SD are shown italicised in brackets).

Superordinate	Utterance type	Mean prop. *(SD)*	Mean raw *(SD)*
Topic-supporting	Contingent Elaboration	0.72 *(0.21)*	25.69 *(15.50)*
Non-reciprocal	Self-contingent Elaboration	0.24 *(0.20)*	11.29 *(11.46)*
Non-reciprocal	Non-contingent Elaboration	0.04 *(0.07)*	1.31 *(2.51)*
	Elaboration – indeterminate	0.00 *(0.00)*	0.02 *(0.14)*

#### Investigating the role of cognitive and socio-cognitive predicators

Following the pre-registered analysis plan, we fitted regression models for each type of elaboration. Here, we deviated slightly from the pre-registration by carrying out mixed-effects logistic regression (and not linear regression) with the target type of elaboration coded as ‘1’ and the other types of child elaboration coded as zero.^6^ For self-contingent elaborations, the only predictor to explain unique variance was working memory (b = −0.50, *SE* = 0.23, χ^2^ = 5.1, p = 0.024), which was negatively related. For contingent elaborations, there was a marginally significant positive relationship with working memory (b = 0.33, *SE* = 0.17, χ^2^ = 3.81, *p* = 0.051).

It is important to highlight the wide individual differences; 19% of children never produced self-contingent or non-contingent elaborations. Nonetheless, indicators of off-topic verbosity were common in these neurotypical 6-year-olds; 25% of children produced self-contingent elaborations in 20% or more of their responses.

### Discussion

There is a great deal of variability among neurotypical, monolingual 6-year-olds regarding if and how they manage to maintain reciprocal conversation. Around a third demonstrate quite a high degree of either non-contingent responding and/or off-topic verbosity. In line with the (relatively scant) literature to date, Study 2 indicates that these individual differences in conversational topic maintenance align with individual differences in theory of mind (De Rosnay et al., 2014; Slomkowski & Dunn, 1996) and working memory (Blain-Brière et al., 2014). Our next study addressed the question of whether a similar pattern of interrelationships is seen for individual differences among autistic children regarding their conversational topic maintenance.

## Study 3

### Method

#### Participants

Participants were scheduled for an Autism Diagnostic Observation Schedule (ADOS) 2 (Lord et al., 2012) module 3 assessment with a clinical psychologist (the 4th author) as part of an NHS multi-disciplinary autism assessment. 50 children were tested (13 female) between January 2019 and March 2020. Of these, 41 received an autism diagnosis and all scored above threshold on the ADOS-2 total algorithm scores (mean score = 12.7). In accordance with the requirements of module 3, part of the eligibility criteria included speaking English fluently. All children indeed spoke fluently in sentences and with southern English accents.

One autistic child did not provide any conversation data during the ADOS-2 and was excluded from analyses. Thus, the final sample comprised 40 children (10 female) aged between 5;4 and 9;10. We had originally aimed to include 60 autistic children (sample size determined by budget) but direct assessment was curtailed due to the outbreak of COVID-19 and first UK national lockdown.

#### Conversation

Conversation was elicited as part of the ADOS-2 module 3 administration which assesses the child’s ability to ‘build on [psychologist’s] statements . . . and take a full role in back-and-forth conversation’. We excluded all verbal interaction that was part of the elicited narratives, make-believe play, interactive play, construction task, interview about friendships and other ADOS-2-related tasks. The administrator always included the majority of the pre-planned statements listed in the supplementary materials. The transcribed conversations were all coded by the same Linguistics Masters student who coded Study 2 using the same criteria (see https://osf.io/q7wa4/ for the coding manual). The first author coded five children (i.e. 755 child utterances) blind to the original coding with good inter-rater reliability (Cohen’s *k* = .872).

#### Additional measures and procedure

After the child had completed the ADOS-2, an RA assessed the child on the following measures in the following order.

*Non-verbal reasoning* was assessed using raw scores on the Matrices sub-test in the Wechsler Preschool and Primary Scales of Intelligence (Wechsler, 2012). Then, we carried out the Backwards Word Span measure of verbal working memory (Blakey et al., 2020). After that, *Expressive Vocabulary* was assessed using raw scores on the CELF-4 (as for Study 2).

Following this, theory of mind was assessed in ‘stepwise’ manner. First, all children were administered two first-order false belief measures (Perner et al., 1987; Wimmer, 1983). If a child passed both, then the RA administered the advanced test items ‘Birthday Surprise’ (Sullivan et al., 1994) and ‘Robots’ (Coull et al., 2006). If, however, the child failed one or both first-order ToM tests, then it was assumed that the latter advanced measures would be failed and instead three measures of ‘precursor’ theory of mind were administered (Pillow, 1989; Wellman & Liu, 2004). In this way, without administering too many tests, it was possible to allocate a child a score on a theory of mind composite, which was then used in analyses.

Finally, the RA administered the Backwards Digit Span from the WISC (Wechsler, 2003). In analyses, for ‘*Working Memory’ we used a composite (mean z scores) of the Backwards Digit raw scores and Backwards Word Span raw scores, which were highly positively correlated (r* (40) = .56, *p* < .001).

All the above predictor variables were significantly positively correlated (see Table 9 Supplemental), with the exception of age, which was only correlated with the vocabulary raw score (*r* (df = 38) = .41, *p* = .01).

### Results

This study was pre-registered (see https://osf.io/q7wa4/registrations) and the same analysis plan employed as for Study 2 with one exception – we did not test inhibitory control. (See Supplemental Table 10 for correlations).

### Child direct responses to experimenter turns

#### Distribution of conversational response types

Table 6 illustrates mean proportions and mean raw frequencies for all coded categories of direct responses to a conversational turn by the ADOS-2 administrator. Due to the NHS ethics required for this study, we did not have access to video-recordings and thus could not code non-verbal responses.

**Table 6.** Study 3 (autistic) child direct response to ADOS-2 administrator turn (mean proportion, mean raw frequency). (SD are shown italicised in brackets).

Superordinate	Utterance type	Mean prop *(SD)*	Mean raw *(SD)*
Topic supporting	Contingent	0.33 *(0.16)*	26.98 *(14.31)*
	Specific Minimal	0.21 *(0.08)*	16.90 *(7.32)*
	Laughing response	0.03 *(0.03)*	2.13 *(2.41)*
*Overall proportion topic-supporting responses to experimenter turns*		*0.57*	*46.00*
	Non-contingent	0.10 *(0.10)*	7.53 *(6.48)*
	Generic minimal	0.19 *(0.11)*	15.88 *(11.48)*
	No audible response	0.14 *(0.14)*	10.88 *(10.25)*

#### Investigating the role of cognitive and socio-cognitive predicators

No predictors explained unique variance in contingent responding. However, as for Study 2, unique variance in all topic-supporting responding was explained by theory of mind (b = 0.38, *SE* = 0.18, χ^2^ = 4.39, *p* = .036). For the non-contingent responses, age was the only predictor to explain unique variance (b = −0.64, *SE* = 0.27, χ^2^ = 5.64, *p* = .018). This was negatively related such that (verbally fluent) autistic children become less non-contingent with age.

### Child elaborations on their own preceding turn

Table 7 indicates the mean proportion and mean raw frequencies of each sub-type of child elaboration. No predictor explained unique variance for any of the elaboration sub-types.

**Table 7.** Study 3 (autistic) child elaboration type (mean proportion, mean raw by child). (SD are shown italicised in brackets).

Superordinate	Utterance type	Mean prop *(SD)*	Mean raw *(SD)*
Topic-supporting	Contingent Elaboration	0.60 *(0.24)*	20.58 *(14.50)*
Non-reciprocal	Self-contingent Elaboration	0.33 *(0.21)*	14.65 *(16.03)*
Non-reciprocal	Non-contingent Elaboration	0.07 *(0.09)*	2.50 *(3.15)*
	Elaboration – indeterminate	0.00 *(0.00)*	0.03 *(0.16)*

#### Individual differences

Around a third of our autistic sample here showed either non-contingent responding to the experimenter or showed self-contingent elaborations (off-topic verbosity) in 20% or more of their utterances. While a very small handful of autistic participants showed fairly extreme tendencies in this regard, at the other extreme, there was also a handful of autistic children who never responded non-contingently nor exhibited any off-topic verbosity.

### Discussion

Compared to study 2, the relationship between the cognitive and socio-cognitive predictors and measures of conversation was less clear. Nonetheless, topic-supporting responding overall was explained by theory of mind as for study 2. Autistic children also became less likely to respond non-contingently with age. Finally, as for Study 2 (with neurotypical children), the autistic children in Study 3 showed great heterogeneity in their conversational behaviours.

## General discussion

We carried out three studies in which we examined conversational skill by categorising children’s turns during live interaction. Study 1 examined conversational differences and similarities between verbally fluent autistic 5- to 7-year-olds and their neurotypical peers. Study 2 investigated relationships between conversation and theory of mind, working memory and inhibitory control in neurotypical 6-year-olds. Study 3 investigated relationships between conversation, theory of mind and working memory within autistic 5- to 9-year-olds.

Our main analyses in all three studies examined direct responses to statements uttered by the conversation partner. We first conflated all responses which supported the topic either by being relevant or by acknowledging the specific content of the immediately preceding turn. However, not all ‘topic-supporting’ responses contribute equally to maintaining a conversation topic; topic-supporting responses include specific minimal responses (such as ‘Wow!) and non-verbal gestures and facial affect responses. While the latter acknowledge the conversation partner’s contribution and encourage him or her to continue, they do not extend a conversation. Therefore, we also investigated ‘contingent’ responses, which by definition keep a conversation going since they elaborate on the topic of the conversation partner’s turn. In terms of responses, which do not support the conversation topic, we focused, first, on ‘non-contingent’ responses, since these are highlighted in the literature as being particularly problematic for conversational back-and-forth (Bloom et al., 1976; Hale & Tager-Flusberg, 2005; Nadig et al., 2010). Second, in Studies 2 and 3 (for which we elicited extended conversation), we also examined ‘self-contingent elaboration’ (Nadig et al., 2010), which is akin to the ‘off-topic verbosity’ (Pushkar et al., 2000; Ruscher & Hurley, 2000).

In Study 1, we found that autistic children were less likely to give non-verbal responses (gestures or facial affect responses) and more likely to respond non-contingently and/or to give generic minimal responses (e.g. ‘mm’ or ‘oh’). This finding is in keeping with the diagnostic criteria for autism (American Psychiatric Association, 2013) and highlights an aspect of difficulty that contributes to wider social-communication differences in autism. However, it is important to stress that the between-group differences in Study 1 suggest a *relative* difficulty with conversation among (verbally fluent) autistic children, rather than an inability to support a conversation topic, with a number of autistic children showing highly skilled conversational turn taking. Indeed, across conversational response types, the evidence from Study 1 points to important similarities, as well as differences, in terms of conversation skills across neurotypical and (verbally fluent) autistic children.

Moreover, although we cannot compare Study 2 and Study 3 directly, it is interesting that in both studies both working memory and theory of mind emerge as correlates of conversational topic maintenance. In Study 2, working memory related negatively to those types of conversational behaviour, which are a hurdle to reciprocal conversation; working memory was related to self-contingent elaboration (off-topic verbosity) and was marginally related to non-contingent responding to statements in regression analyses. Similarly, there were indications in Study 3 that working memory limits may play a role in difficulties in maintaining a conversation topic; although working memory was not a significant predictor of any conversation variables in the main regression analyses (responses to clinician declarative statements), it was correlated (see Appendix 10) and it was a significant positive predictor of contingent responses to the clinician, if we included responses all clinician turns including questions (Appendix 13). The findings for working memory extend those found by Blain-Brière et al. (2014). Furthermore, in Study 2, working memory related negatively to types of utterances that are lengthy but tend to elicit negative social impression ratings (self-contingent elaboration or off-topic verbosity), whereas working memory related positively to those which are lengthy but which do not veer off the original topic (contingent elaboration or ‘general chattiness’). We also found a marginal relationship between working memory and non-contingent responses to the experimenter in Study 2. One interpretation of these findings could be that working memory is required to retain the current conversation topic at the forefront of one’s mind.

Regarding theory of mind, over Studies 2 and 3, this emerged as a significant predictor variable for various conversational response types (when controlling for all the other key variables: age, vocabulary, non-verbal reasoning, working memory and (for Study 2) inhibitory control). While previous studies have shown a relationship between contingent responses in conversation and theory of mind in both neurotypical (Slomkowski & Dunn, 1996) and autistic children (Hale & Tager-Flusberg, 2005), none have shown this relation to be independent of working memory ability. This is the first study to show this and provides support for proposals that an in-depth consideration of the conversation partner’s perspectives is intertwined with conversational proficiency (De Rosnay et al., 2014; Tomasello, 2018). That the pattern of relationships with conversation topic maintenance appears to be similar among autistic and neurotypical children suggests that individual differences in theory of mind and working memory influence conversational ability regardless of diagnostic status.

### Limitations

While the current studies benefitted from eliciting relatively natural, live conversation and a rich, linguistically informed coding scheme, there are a number of limitations. Sample sizes were small for regression analyses. Moreover, some of the cognitive and social-cognitive measures relied on a single test. In addition, stipulations from NHS ethics meant that we were not allowed to store video-recordings of the ADOS and were thus unable to score non-verbal responses for Study 3. Ideally, we would like to have worked with autistic adolescents to discuss the conversation coding scheme.

### Important steps for future studies

To gain a more precise insight into the degree to which autistic and neurotypical child conversation shows similar patterns of relationships to theory of mind and working memory, an important future step for a future study would be to carry out (large scale) individual differences studies of both autistic and neurotypical children in which conversations are elicited in the same manner and in the same settings. Ideally such future studies should also include individual differences measures of both language processing speed and attention (or tendency for distraction). There is evidence both from populations with no diagnoses (Barnett et al., 2022) and autistic children (Howard et al., 2023) that the ability to sustain attention is related to the amount of expressive language produced. Regarding language-processing speed, a number of studies have found a relationship between autism severity and prolonged turn-taking gaps in conversation for both autistic children (McKernan et al., 2022; Parish-Morris et al., 2016) and adults (Ochi et al., 2019). Therefore, the contributions of attention and language processing speed to social conversational ability need exploration.

### Implications

Perhaps the most important finding of the current study is that the conversation skills that are characteristically difficult to deploy for autistic children are also difficult for very many neurotypical children, apparently for similar reasons. Given that conversation skills are part of the primary school curriculum in many countries, this might be taken to suggest that there should be an increased emphasis on empowering mainstream teachers to scaffold and allow practice time for social conversation in classroom settings (Abbot-Smith et al., 2023). The evidence base regarding how to do this effectively is currently incredibly thin. Future research should test the value of different approaches to supporting conversation skill for a diverse range of children. This might include, for example, following up on the finding that theory of mind is related to the ability to maintain a conversation by testing whether actively encouraging consideration of conversation partner’s current mental states (and how to determine/read these) is helpful (Adams et al., 2012; Winner, 2007). Likewise testing ways to mitigate the effects of limited working memory would be worthwhile. All such research should be conducted with teachers and children and would also open the opportunity to talk meta-linguistically about differences in what each of us needs and prefers when having a conversation.

## Conclusion

Although Study 1 found group-level differences between verbally fluent autistic primary-school children and their neurotypical peers in their ability to support and maintain a conversation topic, there were also striking similarities. Moreover, across both Studies 2 and 3, we found relationships between topic-supporting responding and theory of mind, with some evidence for the cost of limited working memory. Continuity across diagnostic groups regarding responses which are not topic-supporting suggests a need for universal support for social conversation skills within schools, particularly since social communication predicts peer relations, emotional stability and behavioural problems (Conti-Ramsden et al., 2019; Law et al., 2014; Roy & Chiat, 2014; Saul et al., 2023).

## Supplemental Material

sj-docx-1-aut-10.1177_13623613241286610 – Supplemental material for Conversational topic maintenance and related cognitive abilities in autistic versus neurotypical children Supplemental material, sj-docx-1-aut-10.1177_13623613241286610 for Conversational topic maintenance and related cognitive abilities in autistic versus neurotypical children by Kirsten Abbot-Smith, Danielle Matthews, Colin Bannard, Joshua Nice, Louise Malkin, David Williams and William Hobson in Autism

## Notes

1. This is a limitation. However, the vast majority of the neuro-typical sample in Study 1 were recruited from and tested in low-mid to low decile schools and thus greater conversational proficiency in the NT group is highly unlikely to be related to higher SES.

2. Following a reviewer’s suggestion, we examined relationships between the composite of these CCC2 subscales and contingent responding and found there was indeed a weak but significant relationship (*r* = .34, *p* = .02).

3. The same pattern was found when each Theory of Mind measure was entered as a separate predictor, whereby age was then marginally significant (b = 0.23, χ^2^ = 3.82, *p* = .051), one ToM measure – Strange Stories was a significant positive predictor (b = 0.29, χ^2^ = 5.78 *p* = .016), but the other ToM measure (Knowledge-Belief) was not significant (*p* = .55).

4. The same pattern of results was found when each Theory of Mind measure was entered separately, namely age was a significant positive predictor (b = 0.43, χ^2^ = 8.37, *p* = 0.004) and the Strange Stories Theory of Mind measure was also a significant positive predictor (b = 0.31, χ^2^ = 4.4, *p* = 0.036), whereas the Knowledge-Belief predictor was not significant (*p* = .38).

5. The same pattern of results was seen when the two Theory of Mind measures were entered as separate predictors whereby here one of the Theory of Mind measures (Knowledge-Belief) then reached significance (b = −0.55, χ^2^ = 10.28 *p* = .0014) and Strange Stories did not (*p* = .35). Working Memory remained as a marginally significant negative predictor (b = −0.26, χ^2^ = 3.52 *p* = .061).

6. A logistic regression was employed as more appropriate to proportions which are bounded by 0 and 1, than linear regression which assumes a continuous scale. However, in fact, we obtain the same pattern of results if we follow the pre-registration exactly and treat self-contingent elaborations as proportion scores (using all elaborations as the denominator) and running multiple linear regression analyses. The same goes for contingent elaborations, although in this case the trend in the direction of a positive significant relationship for working memory is less marked (*p* = .07).
