# Supplementary material for: Conversational topic maintenance and related cognitive abilities in autistic versus neurotypical children
Source: Autism. 2024 Oct 21;29(3):684–97. doi: 10.1177/13623613241286610 (PMC11894863; doi:10.1177/13623613241286610)
Supplement: sj-docx-1-aut-10.1177_13623613241286610 – Supplemental material for Conversational topic maintenance and related cognitive abilities in autistic versus neurotypical children [file sj-docx-1-aut-10.1177_13623613241286610.docx]

**SUPPLEMENTAL MATERIALS / APPENDICES**

Appendix 1: Study 1 Pre-planned conversation probes

ANIMALS SET

1. I really like animals *(This always followed playing a pet shop game)*
2. I have got a pet
3. My pet has a funny name
4. My pet does something really silly

FRUIT SET*(This always followed playing a fruit sorting game – see pic)*
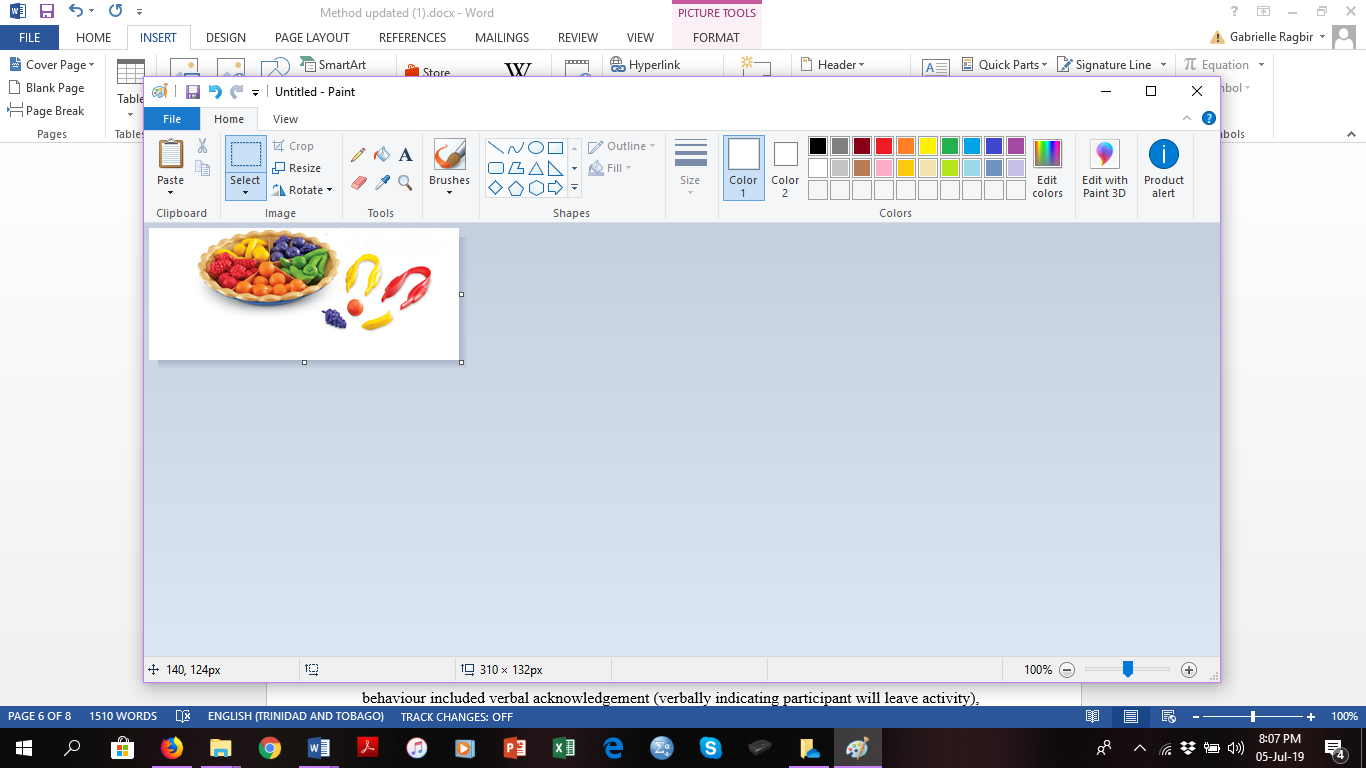


1. I like to eat fruit
2. I think apples are really tasty
3. Some people cook apples
4. I can bake something nice with apples

HOLIDAYS SET*(This always followed the administration of the CELF Expressive Vocabulary – the experimenter pointed to the picture of an island)*

1. Oh look! A beach!
2.
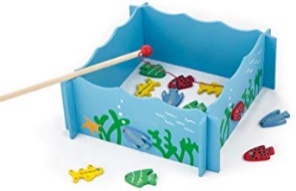
I went to the beach in the summer
3. It was a beach somewhere really special
4. I went on such an amazing trip

GAMES SET*(This always followed a fishing game – see pic)*

1. I really like that game
2. But it’s not my favourite game
3. There’s another game I really like playing
4. The game has a surprise in it

Appendix 2: More detailed description of instructions given to RAs for Study 2

Each child was pre-assigned to one of the two RAs. The children were told that they would carry out some word naming tests, some story tests, some puzzles and a ‘fish’ game but that because some of these were a “bit boring”, they would have some chats in between – and also a ‘biscuit break’ whenever they liked.

During the first ‘chat’, the RA showed the child a picture or object which related to a topic which the child’s parent had named during the recruitment phase as being one of the child’s particular interests. Once conversation was underway, the picture was removed. During the second ‘chat’ break, the picture was of the RA and her brother (as both had brothers) and the elicited conversation revolved around one or more of the following generic topics: siblings, pets and families. The RAs were trained to avoid using an overly questioning style. To aid this, they prepared two or three declarative statements per conversation to use at a topic-appropriate moment. For example, for the ‘swimming’ topic, which was a frequently cited favourite hobby, the RA might prepare a statement about once getting out of her depth before she could swim properly and playing games in the pool. For the sibling topic, the RA usually mentioned her brother once annoyed her in a zoo by pretending to be a monkey and also (at another point in the conversation) mentioned that her brother liked to play tricks on her, such as putting salt in her tea or jumping out and saying ‘Boo!’.

For each of these conversations, the RA was told to aim for around five minutes but not to cut the child off if he or she was (for example) part-way through talking about a topic. The RAs were also told that if it was painfully awkward to continue a conversation, then they could cut it short after three minutes. (This occurred for a few children). Since we used logistic regression with random intercepts for participants, differences between children in conversation length were taken into account.

Example excerpt from conversation (from very proficient child):

**RA Statement**: Once I went to the hospital and they gave me free lollies. [LAUGHS]. They were tasty.

P11: Once I went to a doctor and then they gave me a sweet

**RA Minimal**: Ah! You too!

P11: Well I think it was because of my ears or because of my cough because sometimes I have a cough

**RA Question**: Okay. And you went for your ear because // because it was hurting?

P11: hmm because // because it – no because it – because of the problem with my ears which it doesn’t really hear very well

**RA Statement**: Okay well that’s what doctors are for. They make things better. They help out when // something isn’t working quite well//

P11: [Sneezes]

**RA Minimal**: [LAUGHS] Yeah. Right

P11: Sometimes we – we live inside the [ANONYMISED]

**RA Minimal**: Wow!

P11: And sometimes the police come with their cars – not their motorbikes, though.

**RA Statement**: Hmm – yeah, I saw a policeman on a motorbike once and he fell because he was turning and then he turned too fast and he fell over. Yeah.

P11: And did he hurt himself?

**RA Statement**: Yeah a little bit but I think he got back on the bike quite quickly. I think if you if you get back on it you’re not as scared after.

P11: Well probably not because he wants to finish off his job, doesn’t he?

**RA Statement**: Exactly! He has some people to save so he couldn’t -

P11: - well probably not saved but arrest. [LAUGHS]

Appendix 3: Study 2 (Neurotypical 6-year-olds) Correlation matrix - predictor variable relationships with one another *(N = 48 except where stated)*

|  | Age in months | Expressive  Vocabulary  (raw) | NV reasoning (raw) | WM Backwards Digit Span (raw) | Flanker RT Diff Score (IC) (raw) | Knowledge  & Belief ToM (raw) |
| --- | --- | --- | --- | --- | --- | --- |
| Vocabulary (raw)  (N = 45) | .34* |  |  |  |  |  |
| NVIQ (raw)  (N = 42) | .24 | -.08 |  |  |  |  |
| WM Digit (raw) | .05 | .27 | .09 |  |  |  |
| Flanker RT Diff Score (IC)  (N = 45) | .11 | -.13 | .07 | -.08 |  |  |
| Knowledge  & Belief ToM | -.04 | .34* | .18 | .27"  (*p* = .06) | -.35* |  |
| Strange Stories  ToM | .25 | .17 | .05 | .09 | -.01 | .06 |

" = p < .08 * = p < .05 ** = p < .01 *** = p < .001

Appendix 4: **Study 2 (Neurotypical 6-year-olds) Correlation matrix – proportion of direct responses to all experimenter declarative utterances**

|  | Contingent direct responses | Non-contingent direct responses | All topic-supporting direct responses |
| --- | --- | --- | --- |
| **Age in months**  (N = 48) | .28"  (*p* = .058) | -.12  (*p* = .4) | .16  (*p* .27) |
| **CELF-4 Expressive Vocabulary raw score**  (N = 45) | -.031  (*p* = .84) | .01  (*p* = .97) | -.11  (*p* = .46) |
| **BAS Matrices (NVIQ) raw score**  (N = 42) | .06  (*p* = .7) | -.33*  (*p* = .04) | -.01  (*p* = .3) |
| **WISC Backwards Digit Span raw score**  (N = 48) | -.18  (*p* = .23) | -.15  (*p* = .3) | -.14  (*p* = .33) |
| **Flanker Incongruent – Congruent RT Difference Score**  (N = 45) | .05  (*p* = .7) | .006  (*p* = .97) | .04  (*p* = .79) |
| **Knowledge-Belief (‘Puppy’ + ‘Robots’) ToM raw score**  (N = 48) | .06  (*p* = .70) | -.29*  (*p* = .048) | -.007  (*p* = .96) |
| **Strange Stories (‘Kittens’ + ‘Biscuits’) ToM raw score**  (N = 48) | .21  (*p* = .15) | -.06  (*p* = .69) | .21  (*p* = .15) |
| **Total ToM raw score**  (N = 48) | .17  (*p* = .24) | -.26"  (*p* = .077) | .12  (*p* = .41) |

" = p < .08 * = p < .05 ** = p < .01 *** = p < .001

Appendix 5: Study 2 (Neurotypical 6-year-olds) Correlation matrix – proportion of child elaborations (i.e. child turns which followed on from the child’s own turn)

Elaborations (NB: One child produced no elaborations at all and is excluded from the following analyses)

|  | Contingent elaborations | Self-contingent elaborations |
| --- | --- | --- |
| **Age in months**  (N = 47) | .13  (*p* = .39 | .04  (*p* = .8 |
| **CELF-4 Expressive Vocabulary raw score**  (N = 44) | .21  (*p* = .16) | -.23  (*p* = .14 |
| **BAS Matrices (NVIQ) raw score**  (N = 42) | .22  (*p* = .16) | .11  (*p* = .47) |
| **WISC Backwards Digit Span raw score**  (N = 47) | .33*  (*p* = .024 | -.36**  (*p* = .01) |
| **Flanker Incongruent – Congruent RT Difference Score**  (N = 45) | .09  (*p* = .58) | -.15  (*p* = .34) |
| **Knowledge-Belief (‘Puppy’ + ‘Robots’) ToM raw score**  (N = 47) | .30*  (*p* = .04 | -.31*  (*p* = .03) |
| **Strange Stories (‘Kittens’ + ‘Biscuits’) ToM raw score**  (N = 47) | .06  (*p* = .67) | .01  (*p* = .96) |
| **Total ToM raw score**  (N = 47) | .27"  (*p* = .06) | -.24  (*p* = .11) |

" = p < .08 * = p < .05 ** = p < .01 *** = p < .001

Appendix 6: Study 3 Means (SDs) for age, cognitive and socio-cognitive raw scores

|  | Mean | SE | Minimum | Maximum |
| --- | --- | --- | --- | --- |
| Age in months | 87.12 | 13.62 | 64 | 118 |
| WPPSI Matrices (raw) | 15.10 | 5.54 | 4 | 24 |
| CELF-4 Expressive Vocabulary (raw) | 26.80 | 11.34 | 6 | 52 |
| WISC Backwards Digit Span (raw) | 5.05 | 1.75 | 0 | 9 |
| Backwards Word Span (raw) | 4.78 | 2.22 | 0 | 11 |
| Theory of Mind Composite (raw) | 7.92 | 3.87 | 0 | 13 |

Appendix 7: Study 3 (Autistic 5 to 9-year-olds) Correlation matrix – predictor variable relationships with one another

|  | Age in months | WPPSI  Matrices | Express  Vocab | Backwards Digit Span | Backwards Words |
| --- | --- | --- | --- | --- | --- |
| WPPSI Matrices (raw) | .15 | - |  |  |  |
| CELF-4 Expressive Vocabulary (raw) | .41** | .45*** | - |  |  |
| WISC Backwards Digit Span (raw) | .12 | .33* | .43** | - |  |
| Backwards Word Span (raw) | .29" | .29" | .56*** | .56*** | - |
| Theory of Mind Composite (raw) | .13 | .34* | .69*** | .36* | .57*** |

" = p < .08 * = p < .05 ** = p < .01 *** = p < .001

Appendix 8: Study 3 (Autistic 5 to 9-year-olds) Correlation matrix – proportion of direct responses to all clinician declarative utterances

|  | **Contingent responses** | **Non-contingent responses** | **All Topic-Supporting Responses** |
| --- | --- | --- | --- |
| **Age in months** | -.06  (*p* = .73) | -.3"  (*p* = .062) | -.01  (*p* = .94) |
| **CELF 4 Expressive Vocabulary raw score** | .05  (*p* = .76) | -.16  (*p* = .33) | .23  (*p* = .15) |
| **WPPSI Matrices (NVIQ) raw score** | -.03  (*p* =.84) | .08  (*p* = .63) | .16  *(p = .32)* |
| **WISC Backwards Digit Span raw score** | .19  (*p* = .23) | -.1  (*p* = .56) | .12  (*p* = .46) |
| **Backwards Words Span**  **raw score** | .33*  (*p* = .04) | -.04  (*p* = .83) | .31"  *(p =* .056) |
| **Working Memory composite** | .29"  (*p* = .068) | -.08  (*p* = .65) | .23  (*p* = .15) |
| **Theory of Mind (stepwise) composite** | .24  (*p* = .15) | -.20  (*p* = .21) | .41**  (*p* = .008) |

" = p < .08 * = p < .05 ** = p < .01 *** = p < .001

Appendix 9: Relationship between the ADOS A8 ‘Conversation’ score and our conversation assessment measure

There was a significant negative correlation between the ADOD ‘A8’ (Conversation) score and our direct measure of ‘all topic-supporting’ responding (*r* (36) = -.51, *p* = 0.001) – see figure below. The same relationship was found for contingent responding but the effect size was less (*r* = .34 *p* < .05).

Appendix 10: Study 3 (Autistic 5 to 9-year-olds) Correlation matrix – proportion of child elaborations (i.e. child turns which followed on from the child’s own turn)

|  | **Contingent elaborations** | **Self-contingent elaborations** |
| --- | --- | --- |
| **Age in months** | .17  (*p* = .3) | -.01  (*p* = .96) |
| **CELF 4 Expressive Vocabulary raw score** | .22  (*p* = .18) | .08  (*p* = .64) |
| **WPPSI Matrices (NVIQ) raw score** | .11  (*p* = .51) | .03  (*p* = .87) |
| **WISC Backwards Digit Span raw score** | .32*  (*p* = .045) | .01  (*p* = .93) |
| **Backwards Words Span**  **raw score** | .26  (p = .11) | .13  (*p* = .44) |
| **Working Memory composite** | .33*  (*p* = .04) | .08  (*p* = .64) |
| **Theory of Mind (stepwise) composite** | .19  (*p* = .23) | -.02  (*p* = .89) |

" = p < .08 * = p < .05 ** = p < .01 *** = p < .001

Appendix 11: Study 3 (Autistic 5 to 9-year-olds) Logistic regression - direct responses to all clinical conversational turns (including questions)

Contingent responses

Random effects:

Groups Name Variance Std.Dev.

Participant (Intercept) 0.4498 0.6707

Number of obs: 2612, groups: Participant, **40**

Fixed effects:

B SE Chi-square p-value

(Intercept) -0.50797 0.11624

Age in months 0.04918 0.13374 0.1355 0.7128

CELF -0.19453 0.20099 0.9254 0.3361

NVIQ -0.11931 0.13226 0.8064 0.3692

Working Memory Composite 0.34111 0.16471 4.0815 0.04335 *

ToM_scale_composite 0.12833 0.17062 0.5617 0.4536

Non-contingent responses

Random effects:

Groups Name Variance Std.Dev.

Participant (Intercept) 1.199 1.095

Number of obs: 2612, groups: Participant, 40

Fixed effects:

B SE z value Pr(>|z|) Chi P-val comp

(Intercept) -2.58 0.20 -12.60 < 2e-16 ***

Age in months -0.64 0.23 -2.77 0.006 ** 7.35 0.007 **

CELF 0.17 0.34 0.49 0.62 0.2367 1 0.626

NVIQ 0.23 0.22 1.02 0.31 1.0454 1 0.3066

Working Memory Composite 0.01 0.27 0.04 0.97 0.0016 1 0.9679

ToM_scale_composite -0.30 0.29 -1.02 0.31 1.0023 1 0.3168

All topic-supporting responses

Random effects:

Groups Name Variance Std.Dev.

Participant (Intercept) 0.1792 0 .4233

Number of obs: 2612, groups: Participant, 40

Fixed effects:

B SE z value Pr(>|z|) Chi P-val comp

(Intercept) 0.59 0.08 7.31 2.66e-13 ***

Age in months 0.04 0.09 0.41 0.69 0.1639 0.69

CELF -0.07 0.14 -0.52 0.60 0.2689 0.60

NVIQ 0.06 0.09 0.62 0.54 0.382 0.54

Working Memory Composite 0.07 0.11 0.61 0.54 0.3747 0.54

ToM_scale_composite 0.20 0.12 1.67 0.095 . 2.7193 0.099" .
